# Image Quality of Digital Direct Flat-Panel Mammography Versus an Indirect Small-Field CCD Technique Using a High-Contrast Phantom

**DOI:** 10.4061/2011/701054

**Published:** 2010-10-17

**Authors:** Kathrin Barbara Krug, Hartmut Stützer, Peter Frommolt, Julia Boecker, Henning Bovenschulte, Volker Sendler, Klaus Lackner

**Affiliations:** ^1^Department of Radiology, Medical School, University of Cologne, Kerpenerstraße 62, 50924 Cologne, Germany; ^2^Institute of Medical Statistics, Informatics and Epidemiology, University of Cologne, 50931 Cologne, Germany; ^3^Testing Office for Radiation Protection, Deisterstraße 9, 30974 Wennigsen, Germany

## Abstract

*Objective*. To compare the detection of microcalcifications on mammograms of an anthropomorphic breast phantom acquired by a direct digital flat-panel detector mammography system (FPM) versus a stereotactic breast biopsy system utilizing CCD (charge-coupled device) technology with either a 1024 or 512 acquisition matrix (1024 CCD and 512 CCD). *Materials and Methods*. Randomly distributed silica beads (diameter 100–1400 *μ*m) and anthropomorphic scatter bodies were applied to 48 transparent films. The test specimens were radiographed on a direct digital FPM and by the indirect 1024 CCD and 512 CCD techniques. Four radiologists rated the monitor-displayed images independently of each other in random order. *Results*. The rate of correct positive readings for the “number of detectable microcalcifications” for silica beads of 100–199 *μ*m in diameter was 54.2%, 50.0% and 45.8% by FPM, 1024 CCD and 512 CCD, respectively. The inter-rater variability was most pronounced for silica beads of 100–199 *μ*m in diameter. The greatest agreement with the gold standard was observed for beads >400 *μ*m in diameter across all methods. *Conclusion*. Stereotactic spot images taken by 1024 matrix CCD technique are diagnostically equivalent to direct digital flat-panel mammograms for visualizing simulated microcalcifications >400 *μ*m in diameter.

## 1. Introduction

X-ray stereotactic, ultrasound, or MRI-guided biopsies of the breast have essentially replaced explorative surgical tissue excisions of the breast when findings suspected to be cancer need histological verification. According to the interdisciplinary S3 (level 3) guidelines on the diagnosis, therapy and followup care of the breast cancer issued by the German Cancer Society and its affiliated medical and scientific societies and by the European Commission on quality assurance in screening and diagnosis of breast cancer, the percentage of explorative tissue excisions during screening and diagnostic mammography is reported to be less than 5% [1, 2]. As stated in the guidelines, biopsies should be guided by the technique that in each specific case best visualizes suspiciously malignant findings. 

In most cases, when diagnostic examinations and percutaneous biopsies are performed by ultrasound and MRI mammography, the same system is used, but only with additional stereotactic equipment. Radiologically, by contrast, different systems are usually employed, respectively, for diagnostic imaging and stereotactic biopsy. Before a representative tissue sample can be biopsied for proper pathological testing, the pathological mammographic finding has to be reproduced on X-ray stereotactic spot images. This, in turn, requires that the X-ray stereotactic system offers a spatial resolution and contrast comparable with that of the mammography system. However, it is not a given that the image quality of diagnostic mammograms and X-ray stereotactic spot images are clinically equivalent due to the different detector technologies, imaging geometries, and examination conditions such as the degree of breast compression. 

Since 2003, the authors have been using a direct full-field digital mammography system (Lorad Selenia, Lorad-Hologic, Bedford, MA, USA) together with a digital X-ray stereotactic breast biopsy system (LORAD MultiCare, Lorad-Hologic, Bedford, MA, USA). According to the manufacturer's data, the digital indirect CCD system implemented in the X-ray stereotactic system has a pixel edge length of 50 *μ*m (1024 acquisition matrix), which at least theoretically offers a better spatial resolution than the direct digital flat-panel detector mammography system, with a nominal pixel edge length of 70 *μ*m. Nevertheless, in clinical routine diagnosis, the impression has arisen in isolated cases that the CCD technique was less accurate in detecting smaller microcalcifications than direct digital flat-panel detector mammography was. From March 2003 to June 2009, the two systems were used to perform 807 vacuum biopsies and 487 X-ray stereotactic spring hook markings. In 2008, 127 vacuum biopsies and 135 markings were carried out. The technical success rate of X-ray stereotactic vacuum biopsies was 98.4% for the entire period and 99.2% for 2008; this is comparably high seen against international data [3]. We verify the image quality of simulated microcalcifications in a high-contrast phantom using direct digital flat-panel detector mammography versus a prone stereotactic breast biopsy system with either a 1024 or a 512 acquisition matrix operated according indirect small-field CCD (charge-coupled device) technology. Our primary objective was to establish whether any qualitative or quantitative information is lost when the stereotactic system is operated with the CCD technique. Our secondary objective was to define the minimum size of suspicious lesions on the direct flat-panel detector mammograms that were still detectable by the indirect CCD technique and thereby justify the indication for a biopsy.

## 2. Materials and Methods

### 2.1. Phantom and Simulated Microcalcifications

**Figure 1:**
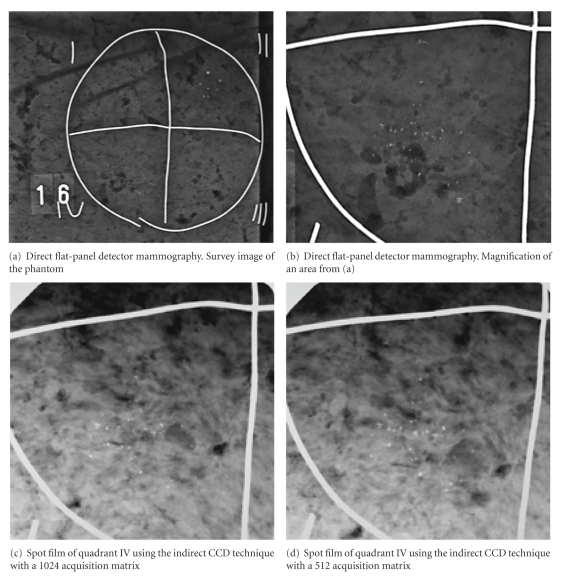
Radiological image of a laser-printer-film covered with different silica beads containing a polymethylmethacrylate (PMMA) sheet of 1.5-cm thickness (Plexiglas, Degussa) and a 1.5-com thick layer of ground meat as scattering bodies. For the raters' orientation, a metal wire was used to divide the phantom into 4 quadrants. Direct flat-panel detector mammography scanned the whole phantom (a). The indirect CCD technique only produced spot images of the phantom's 4 quadrants (c and d). Quadrant IV contains 49 lobular microcalcifications of 300–599 *μ*m in diameter.

A circle of 10 centimeters in diameter was drawn on 48 universal laser printer films with a format of 21 × 30 cm (type A P/N 003R96019, Xerox) and divided into four quadrants. The circles and quadrants were prepared for radiological imaging by labeling them with a metal wire glued onto each transparent film. Next, round or lobular silica beads with a chemical composition of SiO_2_ 65%, Al_2_O_3_ 0.5–2.0%, Fe_2_O_2_ 0.15%, MgO 2.5%, CaO 8.0%, and Na_2_O 14.0%) were scattered randomly on the films within the individual quadrants (Figure 1). The bead diameters were 100–199 *μ*m (class 1), 200–399 *μ*m (class 2), 400–599 *μ*m (class 3), or 600–800 *μ*m (class 4). The number of round and lobular silica beads were counted under a surgical microscope (Wild M680, Leica). 

The 48 films containing simulated microcalcifications were imaged on a digital direct flat-panel detector mammography system (Lorad Selenia, Lorad/Hologic) versus a digital X-ray prone stereotactic breast biopsy system (LORAD MultiCare, Lorad-Hologic). A sheet of 1.5-cm thickness (Plexiglas, Degussa) made of polymethylmethacrylate (PMMA) and a similarly thick layer of ground meat were placed on the films before X-ray exposure to serve as scattering bodies. Both X-ray systems were located in the same room, thereby allowing the images to be produced on the mammography system and the stereotactic breast biopsy system in direct succession without having to change the scattering body configuration.

### 2.2. Imaging Technique

Prior to imaging studies, the two units were serviced by the manufacturer's technicians and the manufacturer's factory-set imaging parameters were optimized by systematic variation. These settings were not changed during the imaging studies. 

The full-field digital mammography system (Selenia, Lorad/Hologic) had a double-focus bimetal anode with a 25-*μ*m molybdenum filter that could be switched to tungsten. The nominal focal spot size for survey mammograms was 0.3 mm and the focus-film distance was 65 cm. The grid was configured like a honeycomb, and because the interseptum material was air, the grid absorbed usable scatter radiation more intensely in all transverse directions. Before X-ray exposure, the semiconductor layer of amorphous selenium on the flat-panel detector was placed under direct current. The X-ray absorption equalized the local charges. The charges were captured behind the selenium layer in an array of electrodes, storage capacitors, and transistors and converted to electronic signals. The imaging data were transmitted to the imaging PC after electronic enhancement and analog-to-digital signal conversion. The active field of view of the flat-panel detector was 24 × 29 cm, the matrix had an array of 3,328 × 4,096 pixels, and the pixel edge length was 70 × 70 *μ*m, equivalent to a nominal spatial resolution of 7.2 lp/mm. Data were acquired in an 18 × 24 cm format. An active field of view of 2,560 × 3,328 pixels was used for imaging. 

The gridless digital prone stereotactic breast biopsy system (LORAD MultiCare, Lorad-Hologic) was equipped with a molybdenum anode with a beryllium port of 0.8 mm and molybdenum filtration of 30 *μ*m in thickness. The nominal focal spot was 0.25 mm, while the focus-film distance was 88 cm. The detector was based on indirect small-field CCD (charge-coupled device) technology. This process involves a scintillator layer of cesium iodide capturing the X-ray quanta and converting them to light rays, which in turn are focused through a lens onto the CCD sensors. The light density distribution was registered as a charge pattern in an array of periodically arranged elements. The sensors are covered by a layer of amorphous silicon where the light quanta are converted to electrical signals and transmitted in local code to the processing computer integrated in the operator console running on Windows NT (Pentium II, Intel Corporation). The active field of measurement was 5.0 cm × 5.0 cm and the pixel edge length 50 *μ*m × 50 *μ*m. In other words, when an acquisition matrix of 1024 × 1024 pixels was selected, a spatial resolution of 10.24 lp/mm was produced and when an acquisition matrix of 512 × 512 pixels was selected, the spatial resolution was 5.12 lp/mm. 

The flat-panel detector mammograms were acquired in the craniocaudal plane, while CCD-based imaging was performed in the ventrodorsal plane. For both procedures, the same compression setting was used in order to eliminate the influence of the degree of compression on image quality. During the data acquisition in the prone stereotactic system, great care was taken to ensure that the vertical arrangement of ground meat layer was also consistently 1.5 cm in thickness. First pretests were conducted on the digital flat-panel detector system to define the optimal exposure stetting: after having varied the tube voltage systematically between 20–32 kV using the automatic exposure control, all mammograms were taken at a tube voltage of 29 kV and tube currents between 23 mAs and 32 mAs. The X-ray stereotactic spot images were taken at 29 kV and oriented along the exposure table with 91 mAs (512 and 1024 acquisition matrix). When the 1024 matrix was selected, the mAs count was not doubled, but rather selected to remain constant. On one hand, this was done to keep the exposure dose within an acceptable range, and, on the other hand, to verify the effect of the higher spatial resolution on the image quality at a constant dose. The surface dose was measured with a calibrated dosimeter (Solidose 300, RTI Electronics AB) at 5.2 *μ*Gy for the direct flat-panel detector mammography, and at 10.5 *μ*Gy for the small-field CCD technique with 1024 and the 512 matrices selected.

### 2.3. Image Display

The mammography system featured a processing station equipped with monitor and PC (Selenia Softcopy Workstation, Lorad/Hologic) running reading software for breast cancer diagnosis (MeVis Breast Care, MeVis, Bremen, Germany). The medical display controller (Barco 5MP1H, BARCO NV, Kortrijk, Belgium) stores the complete 12-bit image on board with 12-bit gray-scale depth (4096 gray levels). A 10-bit digital-to-analog converter (DAC) converted the signal for a display depth equivalent to 1,024 levels of gray. The two Barco monitors used had an image field size of 30 × 40 cm in diameter with a line resolution of 2,048 × 2,560 pixels (effective pixel edge length, 147 × 156 *μ*m). Whenever a scan with a format of 18 × 24 cm was completely displayed on the monitor, this resulted in a factor 0.8 reduction in the geometric resolution to 7 lp/mm. With a 1 : 1 reproduction of the digital image data set, the image was cut off slightly on one edge, producing an effective display resolution of approximately 8 lp/mm.

The stereotactic system featured an operator console equipped with a microprocessor (Pentium II, Intel Corporation) and a 21-inch gray-scale monitor that was used for both image generation and reproduction. The monitor (Model M21L-0213, Image System Corporation) had an image field size of 41 × 31 cm in diameter with a line resolution of 1280 × 1024 pixels (effective pixel edge length of 0.32 mm × 0.3 mm). The monitor's video bandwidth was 200 Hz; the maximum horizontal resolution was 1600 pixels; the maximum vertical resolution 1200 pixels, and the electronic image datasets had a 14-bit gray-scale depth.

On both operating consoles, the brightness and contrast could be changed interactively and their bicubic interpolation allowed factor-2 zooming of the digital image dataset in the sense of an “optical magnifying glass.”

### 2.4. Image Interpretation

**Figure 2:**
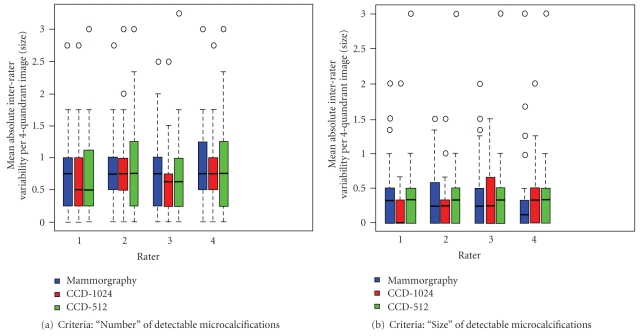
Mean absolute interrater variability of the 4 radiologists' ratings from an experimentally preset reference values for each quadrant of the 48 universal laser printer films are presented here in box plots for the variables “number” and “size” separately for each rater. Statistical outliers are labeled with a dot (•). In the global comparison of all simulated microcalcifications, the diagnostic accuracy of direct digital flat-panel detector mammography system versus the small-field CCD stereotactic breast biopsy system utilizing was comparable, and independently of number, size, or shape of the silica beads.

Four radiologists with 1 to 17 years' experience with analog and 1 to 6 years' experience with digital mammography rated the monitor-displayed digital images independently of each other in randomized order. Before starting their interpretations, the radiologists were given instructions about the image interpretation specific to each system. Their notations were entered on structured electronic questionnaires. The raters were asked to rank the number of simulated microcalcifications visible in each film quadrant according to the following categories: “0" (no microcalcifications), “1" (1–4), “2" (5–9), “3" (10–19), “4" (20–39), or “5" (>39). The size of visible silica beads was rated as “0" (quadrant with no visible microcalcifications), “1" (diameters of 100–199 *μ*m), “2" (200–399 *μ*m), “3" (400–599 *μ*m), or “4" (>600 *μ*m). The shape of visible microcalcifications was classified as as “round," “lobular," or “question not applicable" (quadrants without any silica beads visible). The radiologists classified size and shape based on the largest silica bead visible in the quadrant to be interpreted. For orientation, the raters were given radiographs of silica beads in all occurring sizes and shapes (Figure 2). The rooms were darkened during the monitor readings.

### 2.5. Statistical Analysis

The notations made by the 4 radiologists were divided into imaging techniques as follows: Series 1: digital flat-panel detector mammography, Series 2: CCD technique with a 1024 acquisition matrix, and Series 3: CCD technique with a 512 acquisition matrix. The ratings were then compared with the experimentally preset reference values (gold standard) for the rank-scaled variables “number” and “diameter” and the dichotomous variable “shape.” Next, the data on the four radiologists' correct ratings were cross-tabulated. To measure of how many ratings made by one radiologist per series agreed with the reference standard, the kappa values (*κ*) were calculated, where a negative kappa value or *κ* equal to zero indicated lack of or purely coincidental agreement and a *κ* of 1 indicated full agreement with the reference standard. 

Then, the interrater variability from the reference values per quadrant were determined for the variables “number” and “size” of the detectable microcalcifications to measure the direction and extent of the incorrect interpretations (deviations from the reference values). Under the premise that each of the four quadrants on one film represented an independent observation, each rater made 4 × 48 and 192 allocations, respectively, per series and analysis variable; the statistical analysis of error (interrater variability) was based on these allocations. A rater's variability from the reference values for the variables “size” and “shape” allowed us to determine trends towards underestimations (negative sign) or overestimations (positive sign) in that radiologist's ratings of the findings.

To graphically comparative the interrater variability, the raters' variations for the variables “number” and “size” were presented in box plots and those for “shape” contingency tables according to imaging technique and independently of silica bead size. To describe the effect that size of the simulated microcalcifications had on the sensitivity and accuracy of their detection, the variables “number” and “shape” were each analyzed again separately according to the individual size class of the silica beads. 

The Kruskal-Wallis test was used for global comparison of the results of the 3 imaging techniques. The distribution of interrater variability from the experimentally preset reference values (gold standard) was determined for each imaging technique based on the direction of error (over- or underestimation) and size of the error. The Mann-Whitney test was used for explorative paired comparisons. The *P* values were not corrected for multiple test scenarios. Thus, the interpretation of the results is of a purely explorative nature.

## 3. Results

Table 1 presents the number of raters' correct positive mentions measured against the reference standard. Independently of size, the *number of silica beads* was counted correctly by all raters on 59.3% of the digital mammograms (minimum: 27.7% for ≥40 beads, maximum: 63.9 for 5–9 beads), on 62.0% of the CCD images with a 1024 matrix (minimum: 23.2% for ≥40 beads, maximum: 70.8 for 5–9 beads) and on 60.7% of the CCD images with a 512 matrix (minimum: 17.0% in ≥40 beads, maximum: 66.0 in 5–9 beads) (Table 1(a)). By contrast, the rate of correct positive ratings on the variable “number of detectable microcalcifications” in silica beads between 100–199 *μ*m in diameter (category 1) was 54.2% for digital flat-panel detector mammograms, 50.0% for CCD-1024 images and 45.8% for CCD-512 images. The degree of agreement between the radiologists' ratings and the gold standard (full agreement: Kappa (*κ*) = 1.0, no agreement: *κ* = 0.0) was comparable among the imaging techniques: *κ* was 0.49, 0.53 and 0.50, for digital mammograms, 1024-matrix CCD images, and 512-matrix CCD images, respectively. 

When analyzing all raters and all quadrants independently of the number of silica beads, the *diameter of the simulated microcalcifications* was rated correctly in 74.4% (minimum: 64.7% for 100–199 *μ*m diameters, maximum: 88.0% for 400–599 *μ*m diameters) of the digital mammograms (*κ* = 0.68), in 74.6% (minimum: 64.7% for 100–199 *μ*m diameters, maximum: 91.7% for ≥600 *μ*m diameters) of the 1024-matrix CCD images (*κ* = 0.68) and in 67.1% 512-matrix CCD images (minimum: 50.0% for 100–199 *μ*m diameters, maximum: 97.4% for ≥600 *μ*m diameters) (*κ* = 0.59) (Table 1(b)). By contrast, the radiologists correctly rated the size of detectable microcalcifications for silica beads of 100–199 *μ*m in diameter (category 1) 64.7% of the time on the digital mammograms and the 1024-matrix CCD images, but only 50.0% of the time on the 512-matrix CCD images. 

**Figure 3:**
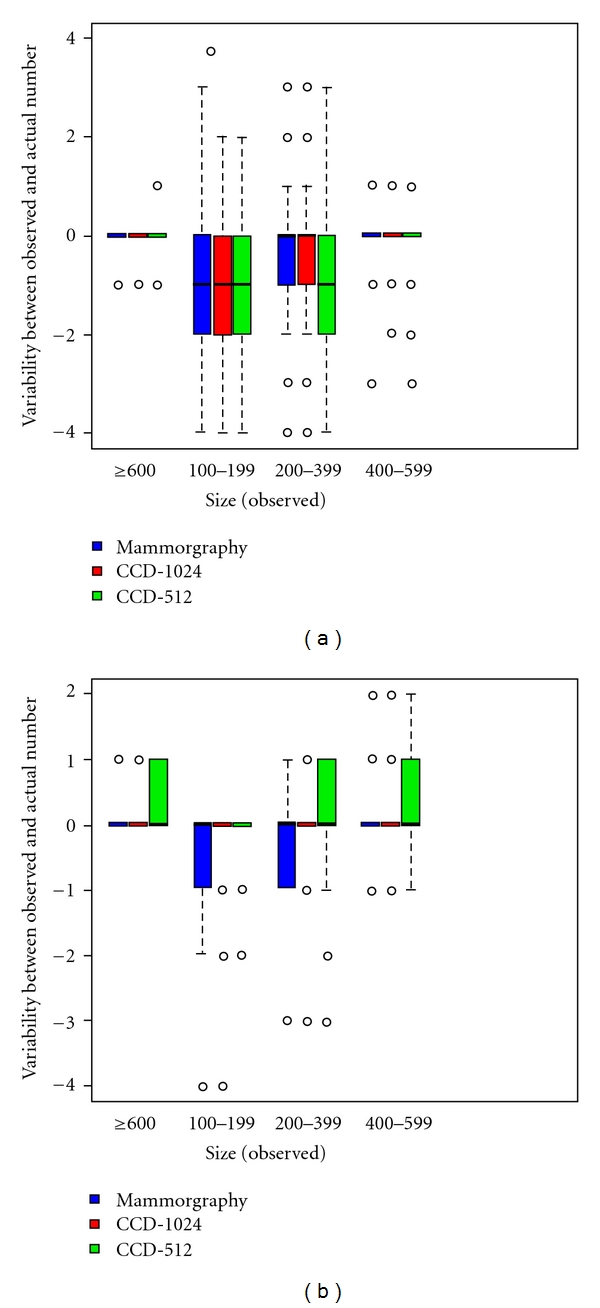
(a) Box plot presenting the interrater variability of the 4 radiologists' from the experimentally preset gold standard as a function of size and number of simulated microcalcifications scattered on the 48 films. Statistical outliers are labeled with an asterisk (∗) or a dot (•). Criterion: “Number" of detectable microcalcifications as a function of the experimentally preset size of silica beads. (b) Box plot presenting the interrater variability of the 4 radiologists' from an experimentally preset gold standard as a function of size and number of simulated microcalcifications scattered on the 48 films. Statistical outliers are labeled with an asterisk (∗) or dots (•). Criterion: “Size” of detectable microcalcifications as a function of the experimentally preset size of the silica beads.

As expected, the rate of correct classifications increased proportional to silica bead diameter. In Figure 3, the results for all quadrants and all raters are presently separately according to the variables “number” (Figure 2(a)) and “size of detectable microcalcifications" (Figure 2(b)) as a function of size categories 1 to 4 measured against the reference standard. While errors in the diagnostic classification of microcalcifications with diameters of 100–199 *μ*m (category 1) showed the greatest variability from the experimentally prescribed reference values, the radiologists' ratings of the size category 3 (diameter 400–500 *μ*m) and 4 (diameters ≥600 *μ*m) largely agreed with the reference values for all three imaging techniques. In size category 1 (diameters 100–199 *μ*m), the number and the size of simulated microcalcifications showed a tendency to be underestimated. In silica beads of size category 2 (diameters 200–399 *μ*m), the raters showed a comparably great uncertainty estimating the number of detected microcalcifications. 

In classifying the shape of the silica beads, the digital mammography tended to be superior to the CCD technique, irrespective of the acquisition matrix used.

The interrater variability from an experimental gold standard was most pronounced for silica beads of 100–199 *μ*m in diameter (Figure 3). The greatest agreement with the gold standard was observed for beads >400 *μ*m in diameter across all methods.

When all imaging techniques were subjected a global comparison by the Kruskal-Wallis test, explorative analysis of the classification errors measured against the experimentally preset gold standard confirmed that there were no remarkable differences between the imaging techniques for the variables “number” (Table 2(a)) or “size of detectable microcalcifications” (Table 2(b)). Explorative analysis using the Mann-Whitney tests on the variable “number” also produced no notable differences between the three imaging techniques (Tables 2(a) and 2(b)). For 2 of the raters, significant differences between the results of digital mammography and those of CCD technique with 1024 and 512 matrix were described for the variable “size” (*P* < .005). However, this result was not confirmed by the data produced by the other two raters.

## 4. Discussion

The technologic demands placed on mammographic X-ray imaging systems are particularly great. First, the dynamic range must be broad enough to enable simultaneous visualization of both structures with strong X-ray absorption, such as calcifications, and those with poor absorption, such as fatty tissue. Second, a comparatively high local image resolution is required to allow detection and morphologic characterization of microcalcifications. The objective of the mammography screening introduced in Germany in 2005 was to detect breast cancer as early as possible when it is in a curable stage. Therefore, it is vital that very small, mammographically detected microcalcifications suspected to be malignant can be stereotactically visualized for percutaneous imaging-guided X-ray biopsy. 

In the past years, different technologies for digital imaging data acquisition have emerged. In general, the following methods are available [4–12]:

- digital storage phosphor radiography;
- digital flat-panel detector radiography with indirect conversion. (Step 1: X-ray quanta captured in a scintillator layer made of gadolinium oxysulfide or cesium iodide are converted to light. Step 2: the light is converted to electrical charges in photodiodes made of amorphous silicon);
- direct flat-panel detector radiography where X-ray quanta are directly converted into electrical charges; and
- a combination slot-scan and photon-counting technique recently introduced on the market.

The study presented herein compared the image quality of an indirect digital small-field CCD detector integrated into an X-ray stereotactic breast biopsy system versus that of a direct digital flat-panel detector of a mammography system. Their imaging geometries differed in terms of imaging data acquisition and imaging data reproduction. Because the CCD system utilized a monocrystalline silicon wafer, the size of the detector was physically and technically limited to an area of 5 × 5 cm. This was compared with a flat-panel detector system with a detector area of 24 × 29 cm. Due to the different clinical targets, the focus-film distances differed: 65 cm for the flat-panel detector versus 88 cm for the CCD technique. The nominal spatial resolution of the acquisition matrix was 7.2 lp/mm, 10.2 lp/mm, and 5.1 lp/mm when the direct flat-panel detector mammography, the 1024 matrix CCD, and the 512 matrix CCD were selected, respectively. The surface dose of 5.2 *μ*Gy with the direct flat-panel detector mammography was lower than the 10.5 *μ*Gy administered with the CCD technique. The manufacturer-stated mathematical pixel edge length on the viewing monitors was 147 × 156 *μ*m for the flat-panel detector and 285 × 185 *μ*m for the CCD technique. The mAs settings were made by automatic exposure control on the flat-panel detector and by an exposure table for the CCD technique, while keeping the kV selection the same. The gray-scale depth was 12-bit and 14-bit for the flat-panel detector and the CCD techniques, respectively. 

The compression setting was kept constant in the phantom study in order to solely visualize the effects of the different equipment techniques on image quality. However, it has to be taken into account that in patient examinations, the degree of breast compression usually differs between diagnostic mammograms and X-ray stereotactic views taken to plan an intervention. Independent of the technology used, lower compression grades are associated with an inferior image quality due to several factors such as a higher amount of scattered radiation, a larger object-to-detector distance of structures located far from the detector surface and the total absorption of low-energy fractions of the radiation spectrum, which otherwise would take part in image contrast. 

The results showed that the X-ray stereotactic spot images of simulated microcalcifications of 100–199 *μ*m in diameter acquired by the CCD technique, especially when the 512 data acquisition matrix was selected, tended to be inferior to the digital direct flat-panel detector technique. Not until diameters >400 *μ*m were the two imaging techniques diagnostically equivalent in detecting simulated microcalcifications.

The quality of a mammographic imaging system is dictated by its capability to convert the radiation pattern behind the breast into a monitor or film image with the highest possible accuracy. The spatial resolution, contrast and signal-to-noise ratio are the key parameters affecting image quality, with spatial resolution and contrast being are mutually dependent upon each other. Raising the spatial resolution while keeping the exposure dose the same leads to a reduction in the photons received in a pixel of the acquisition matrix and thus to a reduction in the contrast resolution and vice versa. The concept of digital quantum efficiency (DQE) was introduced to explain this paradigm. The DQE indicates the percentage of photons hitting the detector that are converted into image data. Most manufacturers list a 50%–90% DQE for digital mammography systems. It is determined by comparing the local dose behind the filters with the exposure dose and represents all steps in the exposure of X-ray radiation up to the radiation pattern being received by the detector. The DQE, however, does not account for the subsequent processing steps (analog-to-digital conversion, technical noise, form of image data processing, etc.), and the quality of the imaging system.

That the direct flat-panel detector mammography produced better images of the simulated microcalcifications <200 *μ*m diameters can be explained by the higher contrast resolution of the digital flat-panel detector and the higher spatial resolution of the viewing monitor. Here, diameters of 130 *μ*m–200 *μ*m are regarded as the lower limit at which simulated microcalcifications can be imaged under in vitro conditions [13–15]. Within the scope of similarly designed in vitro study, Suryanarayanan et al. proved that the performance of a high-spatial-resolution prototype digital imager with pixel sizes ranging between 39–78 *μ*m was superior in terms of detecting microcalcifications when compared with a clinical full-field digital mammography system that yielded a 100-*μ*m pixel size [16]. In their perception study, Rong et al. compared different digital techniques for detecting simulated microcalcifications between 112–160 *μ*m in diameter to find that the spatial resolution and contrast of the monitor was a key factor influencing imaging quality [14].

In general, phantom studies can only give a simplified, more or less one-dimensional simulation of medical reality in all its complexity. Such experiments can hardly reproduce the plethora of technical, biological, pathophysiological, and pathological-anatomical factors impacting diagnostic accuracy along with their mutual interactions. Whereas in-vitro studies cannot predict with certainty the accuracy, a diagnostic imaging technique will produce in clinical examinations, phantom studies do indeed allow an estimation of the diagnostic accuracy that will be obtained during patient examinations. It can be assumed that despite anatomical superimpositions, the microcalcifications as visualized in a phantom will also be reproduced in imaging studies on patients. By the same token, microcalcifications that are undetectable under ideal experimental conditions will presumably not be detected in routine clinical settings either. 

In conclusion, X-ray stereotactic spot images of simulated microcalcifications of 100–199 *μ*m in diameter acquired by the CCD technique, especially when the 512 data acquisition matrix was selected, tended to be inferior to the digital direct flat-panel detector technique. Not until diameters >400 *μ*m did the two imaging techniques produce comparable accuracy in detecting simulated microcalcifications. Consequently, and given the increasing number of suspiciously malignant, small-volume microcalcified foci discovered during mammography screenings, the physical and technical quality of X-ray stereotactic biopsy equipment should be adapted to fit the respective diagnostic digital mammography system.

## Figures and Tables

**Table 1a:** (a) Correct notations as a function of the number of silica beads

Gold standard: number of silica beads [class]	Number of correct ratings [*N* (%)]			Quadrants rated [*N* (%)]
	Digital Mammography	1024 CCD	512 CCD	
0	131 (83.97)	143 (91.67)	152 (97.44)	156 (100.0)
1–4	13 (54.17)	12 (50.00)	11 (45.83)	24 (100.0)
5–9	92 (63.89)	102 (70.83)	95 (65.97)	144 (100.0)
10–19	160 (62.50)	161 (62.89)	164 (64.06)	256 (100.0)
20–39	26 (36.11)	30 (241.67)	23 (31.94)	72 (100.0)
40+	31 (27.68)	26 (23.21)	19 (16.96)	112 (100.0)

Total	453 (59.29)	474 (62.04)	464 (60.73)	764 (100.0)

Kappa	0.49	0.52	0.50	
(*P* value)	<.0001	<.0001	<.0001	

**Table 1b:** (b) Correct mentions as a function of size

Gold standard: diameters of the silica beads [*μ*m]	Number of correct ratings [*N* (%)]			Quadrants rated [*N* (%)]
	Digital Mammography	1024 CCD	512 CCD	

0	118 (62.77)	108 (57.45)	82 (44.62)	188 (100.0)
100–199	88 (64.71)	88 (64.71)	68 (50.00)	136 (100.0)
200–399	153 (78.06)	156 (79.59)	138 (70.41)	196 (100.0)
400–599	81 (88.04)	78 (84.78)	75 (81.52)	92 (100.0)
600+	131 (83.97)	143 (91.67)	152 (1897.44)	156 (100.0)

Total	571 (74.35)	573 (74.61)	515 (67.06)	768 (100.0)

Kappa	0.68	0.68	0.59	
(*P* value)	(<.0001)	(<.0001)	(<.0001)	

**Table 2a:** (a) Number of visible silica beads. The explorative analysis of interrater variability from the experimental gold standard did not produce any significant differences (*P* < .05) between the imaging techniques, neither in the global analysis of the three imaging methods (Kruskal-Wallis Test) nor in the paired comparisons of the two imaging methods (Mann-Whitney Test)

Test	Raters			
	1	2	3	4
Kruskal-Wallis test	.85	.71	.41	.63
Mann-Whitney tests				
1024 CCD^a^ versus 512 CCD	.68	.60	.18	.54
FPM^b^ versus 512 CCD	.59	.41	.41	.72
FPM versus 1024 CCD	.89	.76	.63	.35

^a^CCD, charge-coupled device.

^b^FPM, direct digital flat-panel detector mammography system.

**Table 2b:** (b) Size of simulated microcalcifications. The explorative analysis of radiologists' ratings measured against the experimentally preset reference values produced no global difference between the 3 imaging techniques (Kruskal-Wallis test). Explorative analysis using the Mann-Whitney test for raters 1 and 2 produced notable differences in comparing digital mammograms with the CCD images with 1024 and 512 matrix (*P* < .05), but not for the other two raters

Test	Raters			
	1	2	3	4

Kruskal-Wallis test	**.00**	**.00**	<.31	.41
Mann-Whitney tests				
1024 CCD^a^ versus 512 CCD	**.00**	.05	.22	.75
FPM^b^ versus 512 CCD	**.00**	**.00**	.17	.21
FPM versus 1024 CCD	**.02**	**.00**	.87	.30

^a^CCD, charge-coupled device.

^b^FPM, direct digital flat-panel detector mammography system.
